# Muscle–tendon properties of the female athlete: a comparison between “power modulation” and “energy conservation” training exposures

**DOI:** 10.1038/s41598-026-47580-w

**Published:** 2026-04-24

**Authors:** Scott Newbould, Josh Walker, Alexander J. Dinsdale, Sarah Whitehead, Gareth Nicholson

**Affiliations:** 1 School of Sport and Physical Activity, Sheffield Hallam University, Sheffield, UK; 2 Carnegie School of Sport, Leeds Beckett University, Leeds, UK; 3 Leeds Rhinos Netball, Leeds, UK

**Keywords:** Achilles’ tendon, Patellar tendon, Triceps surae, Strength, Female athlete, Anatomy, Health care, Medical research, Physiology

## Abstract

Muscle–tendon function can be categorised into “energy conservation” or “power modulation” tasks. However, little is known about how female tendons adapt to these different loading demands. This study compared muscle–tendon unit morphological and mechanical properties in athletes who regularly undergo either energy conservation (14 runners) or power modulation (eight netballers) activities. Static ultrasound was used to measure Achilles’ and patellar tendon thickness, as well as the thickness, pennation angle, and fascicle length of the gastrocnemii and vastus lateralis muscles. Achilles’ tendon length and moment arm were also measured. Muscle–tendon mechanical measures included plantar flexion and knee extension isometric strength, and Achilles’ tendon elongation, strain, and stiffness. Netballers displayed significantly higher absolute tendon thickness (*p* = 0.004 for Achilles’, *p* = 0.021 for patellar), but not when normalised to body mass. Netballers also displayed significantly higher Achilles’ tendon stiffness (*p* = 0.046) and isometric plantar flexion and knee extension strength (both *p* < 0.001). No differences were detected in any other measure of muscle or tendon morphology. The results of this study provide evidence that power modulation activities promote different muscle–tendon unit properties to energy conservation activities in females, but that these adaptations may be body mass dependent.

**Supplementary Information:**

The online version contains supplementary material available at 10.1038/s41598-026-47580-w.

## Introduction

Lower-limb muscle–tendon units (MTUs) like the triceps surae are key structures in human movement, and their function can be categorised into three roles: “energy conservation”, “power amplification”, and “power attenuation”^1^. Energy conservation strategies are typically seen in endurance activities that are characterised by repetitive submaximal loading cycles where metabolic economy is important, such as long-distance running. In contrast, power amplification and power attenuation strategies (together, “power modulation”) are common in sports where athletes frequently undergo maximal acceleration and deceleration, jumping and landing, and changes of direction.

The magnitude of Achilles’ tendon (AT) strain differs between energy conservation and power modulation tasks, with reported peak strains of less than 6% during running^2^, but over 8% during single leg hopping^3^. Consequently, these different movement demands might result in divergent adaptations to the MTU. This is because the key factor in tendon adaptation is the strain magnitude, which must be sufficient to stimulate adaptations^4,5^. Indeed, a recent meta-analysis of training studies concluded that aerobic training (i.e., energy conservation) in isolation had no effect on tendon properties, whereas jump-based plyometric training (i.e., power modulation) resulted in significant tendon stiffness increases^6^. Tendon adaptation can occur through changes to the tendon’s material (i.e., elastic modulus), morphological (i.e., cross-sectional area, CSA), or mechanical (i.e., stiffness) properties^7^. These aspects are interrelated, as for example an increase in CSA will reduce the stress experienced by the tendon and therefore the magnitude of elongation in response to a constant tensile load, modulating the mechanical properties of the tendon^7^.

Importantly, whilst material changes can significantly alter tendon stiffness within 8–12 weeks of loading^8^, only small and inconsistent changes in CSA are seen over this time frame in training studies^6^. In contrast, a larger AT CSA has consistently been reported in male athletes who regularly undergo both power modulation^9,10^ and energy conservation loading^10–13^. Tendon CSA may therefore be a sensitive marker of adaptation, but because of the relatively slow turnover of tendon tissue^14^, the short-term nature of training studies limits their ability to examine the long-term (i.e., years) effects of tendon loading^15^. Therefore, much can be learned about long-term tendon adaptation from cross-sectional studies such as those which compare MTU properties between populations who have experienced different long-term loading demands. The measurement of tendon properties has implications for athlete performance, as previous research has highlighted the relationships between tendon mechanical properties and athletic performance outcomes such as jump and sprint ability^16–18^ and long-distance running performance^19,20^. Furthermore, the uniformity of muscle and tendon mechanical properties (i.e., muscle strength and tendon stiffness) is important not only for enhancing athletic performance potential, but also for mitigating injury risk^21,22^.

Despite the utility of these measurements, there is currently a scarcity of data in females, which is of concern because of the known differences in tendon properties between sexes^23–25^. These differences might be explained by different acute responses to loading between sexes, whereby females display an attenuated response to exercise^13^. This is possibly because of higher oestrogen levels, which have been shown to reduce markers of positive tendon adaptation after loading^13,26^. Indeed, whilst male runners display different tendon properties compared to untrained controls, female runners do not^13,27^. Although this suggests that female tendon properties might not adapt in response to loading, studies by Vikmoen et al.^28^ and Dalgaard et al.^29^ showed increased patellar tendon (PT) CSA following heavy resistance training (i.e., a high tendon strain activity). As such, the findings from Magnusson et al.^13^ and Westh et al.^27^ might be because running (i.e., a lower tendon strain activity) is a loading type that is insufficient to stimulate adaptations in females.

In contrast, Hansen et al.^30^ found that the jumping leg of female handball players displayed significantly greater PT CSA and stiffness compared to the non-jumping leg, evidencing that chronic power modulation loading stimulates adaptation in females. However, this study did not measure any aspects of the AT, and thus even though power modulation MTU strategies are common to nearly all sports, the long-term response of the AT to this type of loading remains unknown. Additionally, whilst Fletcher et al.^31^ reported differences in the mechanical profile of the AT between male and female runners and Magnusson et al.^13^ and Westh et al.^27^ found no difference in AT morphology between female runners and controls, no study has reported the mechanical and morphological properties together in a female-specific running cohort. In conclusion, the chronic adaptations to these different loading demands in female athletes remains unclear, and therefore the aim of this study was to compare the lower-limb MTU profiles of female energy conservation and power modulation athletes, with measures including strength, morphological characteristics, and mechanical properties.

## Results

### Anthropometrics and muscle morphology

There were differences in stature (runners: 169.0 ± 4.9 cm, netball: 180.0 ± 7.8 cm, *p* < 0.001, *large* effect size 1.75) and body mass (runners: 63.4 ± 5.9 kg, netball: 81.1 ± 7.6 kg, *p* < 0.001, *large* effect size 2.62) between groups. There were no differences in any measure of muscle morphology between groups (*p* ≥ 0.05) (Table 1).

**Table 1:** Descriptive statistics and paired t-test differences between groups for muscle morphology.

	Variable	Runners (Mean ± SD)	Netball (Mean ± SD)	*p*	Hedges’ *g* (95% CI)
Gastrocnemius medialis	Thickness (mm)	18.1 ± 2.7	20.3 ± 2.5	0.077	0.80 (− 0.84–1.66)
	Thickness/shank length	0.042 ± 0.07	0.043 ± 0.006	0.656	0.13 (− 0.72–0.96)
	Pennation angle (°)	17.5 ± 1.8	19.2 ± 1.8	0.050	0.89 (− 0.01–1.76)
	Fascicle length (mm)	63.3 ± 8.7	68.2 ± 9.1	0.225	0.53 (− 0.32–1.38)
	FL/shank length	0.147 ± 0.021	0.144 ± 0.020	0.702	− 0.17 (− 1.00–0.67)
Gastrocnemius lateralis	Thickness (mm)	13.8 ± 2.5	15.3 ± 1.6	0.142	0.65 (− 0.22–1.50)
	Thickness/shank length	0.032 ± 0.006	0.032 ± 0.004	0.784	0.05 (− 0.78–0.89)
	Pennation angle (°)	11.7 ± 2.5	13.3 ± 1.8	0.126	0.68 (0.19–1.54)
	Fascicle length (mm)	64.5 ± 12.7	66.3 ± 7.1	0.727	0.15 (− 0.69–0.99)
	FL/shank length	0.150 ± 0.027	0.139 ± 0.016	0.343	− 0.41 (− 1.25–0.44)
Vastus lateralis	Thickness (mm)	22.0 ± 3.0	24.6 ± 3.1	0.071	0.81 (− 0.07–1.68)
	Thickness/thigh length	0.053 ± 0.008	0.054 ± 0.007	0.682	0.10 (− 0.74–0.94)
	Pennation angle (°)	16.0 ± 2.4	16.6 ± 1.5	0.574	0.24 (− 0.60–1.08)
	Fascicle length (mm)	78.7 ± 9.7	82.2 ± 9.8	0.423	0.35 (− 0.50–1.19)
	FL/thigh length	0.192 ± 0.026	0.180 ± 0.022	0.378	− 0.39(− 1.22–0.46)

SD, standard deviation; CI, confidence interval; FL, fascicle length

### Tendon thickness

**Fig. 1:**
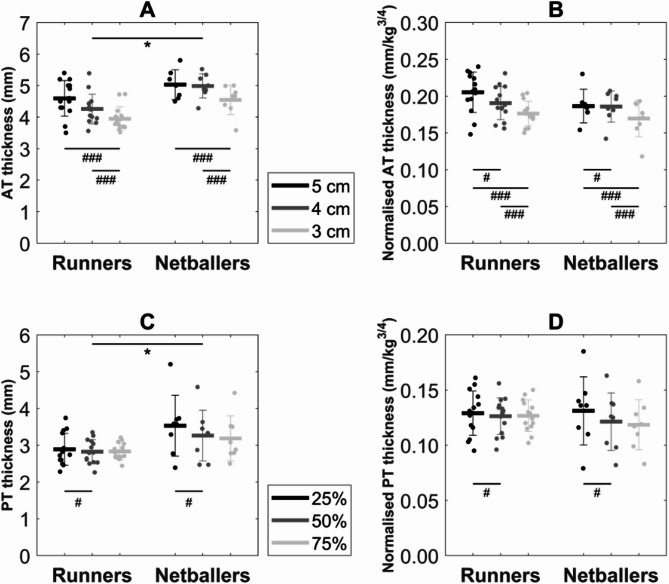
Regional tendon thickness for (**A**) Achilles’ tendon (**B**) normalised Achilles’ tendon, (**C**) patellar tendon, and (**D**) normalised patellar tendon. * denotes a significant difference between groups (*p* < 0.05). # and ### denote a significant difference between regions of *p* < 0.05 and *p* < 0.001, respectively. Between-group comparison of Achilles’ and patellar tendon thicknesses resulted in *large* Hedges’ *g* effect sizes of 1.38 and 1.07, respectively.

A mixed analysis of variance (ANOVA) for AT thickness revealed there was a main effect of group (*p* = 0.010), whereby netballers had a thicker AT than runners (netballers: 4.82 ± 0.44 mm, runners: 4.27 ± 0.42 mm) (Fig. 1A). There was also a main effect of region where AT was thicker at 5 cm (4.81 ± 0.57 mm) and 4 cm (4.60 ± 0.47 mm) than 3 cm (4.21 ± 0.43 mm, both *p* < 0.001) (Fig. 1A). There was no group × region interaction (*p* = 0.373). A mixed ANOVA for normalised AT thickness revealed no difference between groups (*p* = 0.243), but a significant main effect of region (*p* < 0.001), where *post-hoc* testing revealed 5 cm (0.196 ± 0.03 mm/kg^3/4^) was thicker than 4 cm (0.186 ± 0.0.02 mm/kg^3/4^, *p* = 0.041) and 3 cm (0.171 ± 0.0.02 mm/kg^3/4^, *p* < 0.001), and 4 cm was thicker than 3cm (*p* < 0.001) (Fig. 1B). There was no group × region interaction (*p* = 0.151).

PT thickness had a main effect of group (*p* = 0.027), where netballers (3.33 ± 0.46 mm) displayed a thicker PT than runners (2.84 ± 0.46 mm) (Fig. 1C). The ANOVA also revealed a main effect of region (*p* = 0.024), where the 25% region (3.21 ± 0.61 mm) was thicker than the 50% region (3.04 ± 0.50 mm, *p* = 0.025) (Fig. 1C). There was no group × region interaction (*p* = 0.117). A mixed ANOVA for normalised PT thickness revealed no main effect of group (*p* = 0.692), but a significant main effect of region (*p* = 0.008), with *post-hoc* tests revealing that the 25% region (0.132 ± 0.02 mm/kg^3/4^) was thicker than 50% (0.124 ± 0.02 mm/kg^3/4^, *p* = 0.015) (Fig. 1D). There was no group × region interaction (*p* = 0.104).

Pearson’s correlations revealed significant positive *moderate* relationships between body mass and average AT thickness (*r* = 0.482, *p* = 0.027) and PT thickness (*r* = 0.440, *p* = 0.041).

### Muscle–tendon unit mechanical properties

As displayed in Table 2, netballers displayed higher maximal plantar flexion and knee extension joint moments (*p* < 0.001), as well as higher AT force (*p* < 0.001) and AT stiffness index (AT*k*_index_; *p* = 0.046). There were no differences in gastrocnemius medialis (GM) tendinous tissue elongation, strain, AT length, or moment arm between groups (*p* > 0.05). There was no relationship (*p* > 0.05) between body mass and AT*k*_index_.

**Table 2:** Descriptive statistics and paired t-test differences between groups for maximum voluntary isometric contraction moments and Achilles’ tendon properties.

Variable	Runners (Mean ± SD)	Netball (Mean ± SD)	*p*	Hedges’ *g* (95% CI)
Plantar flexion MVC (N.m)	123.1 ± 15.9	175.9 ± 22.4	< 0.001***	2.75 (1.55–3.93)
Normalised plantar flexion MVC (N.m/kg^3/4^)	5.5 ± 0.6	6.5 ± 0.9	0.002**	1.45 (0.49–2.38_
Knee extension MVC (N.m)	133.4. ± 27.7	199.7 ± 32.7	< 0.001***	2.16 (1.07–3.21)
Normalised knee extension MVC (N.m/kg^3/4^)	5.9 ± 1.1	7.4 ± 0.8	0.005**	1.35 (0.40–2.28)
GM tendinous tissue elongation (mm)	12.6 ± 3.4	15.9 ± 4.8	0.074	0.81 (− 0.08–1.67)
GM tendinous tissue strain (%)	6.5 ± 1.7	7.3 ± 1.9	0.344	0.41 (− 0.44–1.25)
Achilles’ tendon force (N)	4171 ± 622	5949 ± 743	< 0.001***	2.55 (1.40–3.70)
AT*k*_index_ (N/strain)	677 ± 177	854 ± 207	0.046*	0.91 (0.02–1.78)
Achilles’ tendon length (mm)	193.4 ± 20.8	216.3 ± 37.3	0.143	0.79 (− 0.09–1.66)
Achilles’ tendon length/shank length	0.448 ± 0.036	0.453 ± 0.069	0.790	0.12 (− 0.72–0.95)
Achilles’ tendon moment arm (mm)	29.7 ± 2.7	29.6 ± 1.9	0.951	− 0.26 (− 0.86–0.81)

SD, standard deviation; CI, confidence interval; MVC, maximum voluntary contraction; GM, gastrocnemius medialis; AT*k*_index_, Achilles’ tendon stiffness index. *, **, and *** denote a significant difference between groups at levels of *p* < 0.05, *p* < 0.01, and *p* < 0.001, respectively.

## Discussion

The aim of this study was to compare the lower limb MTU profiles of female energy conservation athletes (runners) and power modulation athletes (netballers). Netballers possessed higher isometric strength and AT stiffness, as well as a thicker AT and PT, compared with runners. No differences were present between groups for normalised tendon thicknesses, or any other measure of MTU morphology. These findings provide insight into the effects of these different long-term loading demands on the structure and function of the triceps surae and quadriceps muscle–tendon complexes. These data can be used to inform sport-specific training practices for optimising MTU properties in female athletes.

Netballers displayed a thicker AT and PT than runners (*large* effect sizes). This is likely because the power modulation activities such as jumping and landing present in netball^32^ generally result in larger tendon strains than running^33^ and therefore are more stimulative to the tendon^4^. Indeed, whilst Hansen et al.^34^ found that 9 months of running training was not sufficient to change AT CSA, studies by Houghton et al.^35^ and Bohm et al.^36^ reported increases in male AT size as a result of plyometric (i.e., power modulation) training. In addition to the power modulation loading experienced in netball training and competition, the netballers completed 2.5 ± 0.5 strength and conditioning sessions per week (compared to 0.7 ± 0.7 for the runners), which included a combination of plyometric and resistance training. Resistance training is an effective stimulus for tendon adaptation^6^, and the increased muscle strength developed through this type of training means that the netballers’ tendons are likely exposed to greater strains from more forceful muscle contractions during movement^21^. Therefore, even though the volume of tendon loading of the runners is likely higher than that of the netballers in terms of the number of loading cycles and the integral of strain over time, the difference in strain magnitude experienced by these two groups is likely the primary cause of the divergence in tendon size and stiffness.

However, it must be noted that no differences were found between groups for normalised thickness (*p* = 0.320 and 0.697 for AT and PT, respectively). This questions whether the thicker tendons in netballers are caused by loading demands alone, or in conjunction with body mass, as a higher body mass will necessarily place higher forces on the tendon during weight-bearing activities. To further investigate this, correlation analyses were carried out between body mass and absolute tendon thickness metrics, which found *moderate* positive relationships, showing that body mass also influences tendon size, which has been reported previously in males^37^. Tendon size (both absolute and normalised) tended to be greater at the more proximal measurement region for both tendons (Fig. 1). Although speculative, this might be because tendon strain is not uniform across the length or depth of the tendon (e.g.,^38,39^), which results in non-uniform adaptations. Interestingly, no differences were observed between groups for any measure of muscle morphology, meaning that all morphological differences between groups were present in the tendons, rather than muscle. Ultimately, it appears that the combination of loading demands, body mass, and muscle strength all expose the netballers’ tendons to higher relative load and strain, and although caution is warranted because of the cross-sectional nature of the study and the use of thickness measurement rather than CSA, these findings suggest that increased mechanical loading can result in a hypertrophic adaptation to the AT and PT in females.

In the present study, netballers displayed significantly higher AT stiffness than runners (*large* effect size of 0.91). Similarly, Arampatzis et al.^40^ found that male sprinters had greater AT stiffness than endurance runners, demonstrating that power modulation loading results in divergent stiffness adaptations to energy conservation. In further support of this, power modulation activities that cause high tendon strains, such as plyometric training, generate positive AT stiffness adaptations^41^. Thus, the present results provide initial non-causal evidence that female athlete tendons adapt to power modulation-type loading in similar ways to male tendons; future research should investigate this using intervention designs.

The triceps surae muscle strength of an individual might also contribute to increased stiffness, as when reporting data from several of their studies, Arampatzis et al.^21^ showed a significant relationship between the AT force experienced during a maximal voluntary isometric contraction and AT stiffness (*r* = 0.67, *p* < 0.001). This is because more forceful contractions from the triceps surae induce higher AT strains, promoting stiffness adaptation^4^. Although no relationship was found between AT force and stiffness in the present study, the higher AT stiffness in the netballers allowed AT strain to remain within optimal ranges despite higher muscle strength, meaning that functional MTU uniformity between muscle strength and tendon stiffness is maintained^21^. The higher AT stiffness, coupled with the thicker tendons in the netballers, implies that the higher stiffness is because of morphological adaptation, rather than material changes, although this remains somewhat speculative. This is because a thicker tendon will be a stiffer one, assuming a consistent material profile^42^. This adaptation enhances both the structural and functional integrity of the tendon, showing for the first time that power modulation training promotes AT adaptation in female athletes.

Netballers displayed significantly higher isometric plantar flexion and knee extension moments than runners (*large* effect sizes of 2.75 and 2.16, respectively), a difference which might be explained by the resistance training undertaken by the netballers. However, given that resistance training variables (e.g., exercise selection, volume, intensity) were not collected for this study, this supposition cannot be fully supported. Nevertheless, another potentially contributing factor is that in power modulation movements, the MTU is required to rapidly generate and attenuate high forces^1^, which might result in positive strength adaptation. Indeed, power modulation loading has been shown to positively impact muscle strength in plyometric training studies^41^. The higher body mass of the netballers might contribute to the strength difference, as the relevant musculature is required to produce greater forces to generate movement and will be exposed to greater loads during weight-bearing activities. Furthermore, muscle strength is positively associated with better performance and lower injury risk in power modulation activities (i.e., jumps, sprints, change of direction activities^43^, and although strength is also positively related to running economy^44^, the relatively lower forces experienced in energy conservation activities might not be sufficient to stimulate substantial strength adaptations, unlike power modulation activities.

It must be acknowledged that the sample size of the present study was relatively small which reduces statistical power. Nevertheless, significant differences were reported for several of the variables of interest (i.e., muscle strength, tendon thickness and stiffness), indicating that the study was adequately powered to detect these differences between groups. However, these were all differences with *large* effect sizes, and thus it could be the case that there might have been *small* or *moderate* differences between groups in other variables that were not statistically significant because of the sample size. Regardless, the present work has identified differences between these female athlete cohorts for the first time for tendon stiffness and thickness. The measurement of tendon thickness as opposed to CSA is another point for consideration; whilst ultrasound-measured tendon thickness is a reliable metric^45^, it is ultimately a proxy measure for tendon size and precludes the calculation of tendon stress and Young’s modulus. The exclusion of these measurements means the material properties of the tendon have not been measured in this study and instead must be inferred from the relationship between mechanical and morphological properties.

In conclusion, netballers displayed different mechanical function (greater AT stiffness and isometric strength) compared to runners, and these differences were underpinned by morphological differences in the tendons (greater absolute tendon size) rather than muscle. These divergent properties are likely an adaptation to the combination of the increased body mass and the distinct loading demands experienced by the netballers, which place relatively greater forces on the MTU than those experienced by the runners. The present study provides insight into the MTU adaptations of power modulation and energy conservation athletes. Whilst the cross-sectional design provides the benefit of investigating long-term loading, it does not provide control over the exact loading demands (e.g., intensity, volume, concurrent training), or over possible selection bias. Therefore, results should be interpreted within the context of the cross-sectional design of this study. Longitudinal studies are required to confirm the impact of each type of loading on MTU adaptations.

These findings provide evidence that female tendons are responsive to mechanical loading in the form of power modulation type movement. Although previous research has called into question the adaptability of female tendons to loading^13,27^, the results of this study and a recent training study^28^ suggest that the lack of response in the previous investigations is because energy conservation loading is an insufficient stimulus for tendon adaptation in females. The tendon properties observed in the power modulation athletes in this study evidence adaptations that are theoretically beneficial for both performance improvement and injury risk reduction in this population, with uniformity of the muscle–tendon unit being maintained^21^. Therefore, practitioners can use power modulation movements (e.g., plyometric training) to target favourable tendon properties in female power modulation athletes. The results of this study have widespread implications because of the large number of sports where the loading demands or training practices are characterised by power modulation type movements.

## Methods

### Participants

A convenience sample of 22 healthy females took part in this between-group cross-sectional study, which included 14 long-distance runners to represent energy conservation loading (age: 28.6 ± 7.0 years) and eight netballers for power modulation loading (age: 25.2 ± 4.1 years). Netballers were chosen to represent power modulation loading because of the high frequency of jumps, accelerations, and deceleration events present in netball training and competition^32^. Netballers had a total netball training age of 14.1 ± 5.5 years, including 5.2 ± 3.1 years at their current standard, and completed 5.9 ± 0.6 training sessions per week. Runners trained for and competed in races ranging from 5 km to marathon distances, had 7.9 ± 4.9 years of running training, including 6.6 ± 4.6 years at their current standard, and completed 5.1 ± 2.3 running sessions per week for a distance of 62.8 ± 21.9 km/week. Participants were required to be free of any musculoskeletal injury for at least 6 months, as well as never previously rupturing or having tendinopathy of their AT or PT. All participants provided their written informed consent to take part in the study, and the study was approved by the Leeds Beckett University Ethics Committee (approval number 102194) and conformed to the Declaration of Helsinki^46^.

### Procedures

Muscle and tendon morphology were assessed via static longitudinal B-mode ultrasound using a Siemens Acuson P300 system (Siemens Healthineers AG, Erlangen, Germany). The AT and PT were scanned using a 40-mm linear array probe (12–18 MHz), and the gastrocnemius medialis and lateralis and the vastus lateralis muscles with a 50-mm linear array probe (5–12 MHz). All images were analysed using ImageJ software (ImageJ2 1.54h, 64-bit, National Institutes of Health; Bethesda, MD, USA). AT thickness was measured 3, 4, and 5 cm proximal to the calcaneal notch, as this region has been shown previously to differentiate between athlete cohorts^11,12^. Similarly, as regional adaptations to the PT have been shown^9^, PT thickness was measured at approximately 25, 50, and 75% of tendon length^47^, where PT length was defined as the distance from the origin of the PT on the apex of the patellar to the insertion on the tibial tuberosity. Importantly, as tendon loading is impacted by body mass and a significant difference in body mass was present between groups (*p* < 0.001), tendon thickness values were also normalised to body mass to the power of 0.75^9^. Tendon thickness was reported for each location individually, as well as an average of the three locations for each tendon.

For the AT and gastrocnemii scans, participants laid prone on a physiotherapy bed with their ankle fixed at 90° (neutral) and knee fully extended. The ankle was fixed at 90° by using an elastic bandage to secure the participant’s foot to the physiotherapy bed support, and the angle was confirmed using a handheld goniometer (Jamar, UK). For the vastus lateralis scan, participants sat on the bed with their knee fully extended and with a hip angle of 120° (180° in anatomical position), achieved by manipulating the backrest of the physiotherapy bed. For the PT scan participants sat upright with a knee angle of 90°, achieved by having participants sit with their legs off the edge of the bed, with their feet resting on rubber mats which were stacked to a height that resulted in a 90° knee angle (confirmed with a goniometer). The gastrocnemii were scanned at approximately 30% of shank length (distance between lateral femoral epicondyle and lateral malleolus) and the vastus lateralis at approximately 50% of thigh length (distance from femoral greater trochanter to lateral femoral epicondyle). From each muscle scan, muscle thickness (distance between aponeuroses), pennation angle (angle of fascicle relative to deep aponeurosis), and fascicle length (straight-line distance between a fascicle’s insertion at deep and superficial aponeuroses) were measured. If the field-of-view was insufficient to measure a full fascicle, the manual linear extrapolation method was used^48^. Finally, the AT resting length was measured with a tape measure (guided with ultrasound) from the most distal point of the GM myotendinous junction to the calcaneal notch. Muscle thickness, fascicle length, and AT length were also normalised to their respective segment lengths to remove any scaling effect of stature on outcomes^49,50^.

Mechanical properties of the AT were assessed using ultrasound and dynamometry. Participants were seated on a dynamometer (CSMI, Cybex Humac Norm, Stoughton, MA, USA) with a hip joint angle of 60° flexion, the knee in full extension, and an ankle joint angle of 90° (neutral). A 60-mm, 128-element linear array ultrasound probe recording at 15 Hz (LV7.5/60/128Z-2, 5–8 MHz; EchoBlaster 128 CEXT-1Z, Telemed UAB; Vilnius, Lithuania) was placed over the GM myotendinous junction to measure displacement throughout all ankle dynamometry protocols. Participants performed 5-s graded isometric contractions with 20-s rest periods, starting at 20 N.m and increasing by 10–20 N.m until a maximum was reached. Three to five maximal attempts were performed, with the average of these being used for analysis^51^. The displacement of the GM myotendinous junction from rest to the isometric portion of each contraction includes elongation of both the GM aponeurosis and the AT, with the displacement of this landmark being used as the measure of GM tendinous tissue elongation. Throughout all dynamometry procedures, participants were securely strapped into position to minimise joint rotation, but the measured GM tendinous tissue elongation was corrected for unavoidable joint rotation^40^. AT moment arm was calculated using a passive rotation protocol and the tendon excursion method^52^. From these procedures, the variables of interest were the maximum GM tendinous tissue elongation, strain (elongation relative to resting length), AT force (calculated as the maximum joint moment recorded by the dynamometer divided by the AT moment arm), and stiffness index (AT*k*_index_). AT*k*_index_ was calculated by dividing the maximum tendon force by the maximum GM tendinous tissue strain^51^; this metric has previously been shown to display high reliability within a female athlete population^53^, and because its calculation utilises strain rather than elongation, it partitions out the between-subject variability in tendon length. Additionally, participants performed three maximal isometric knee extension trials of 5 s each with 30 s of rest whilst seated on the Cybex dynamometer with a knee joint angle of 90° and a hip joint angle of 80° flexion. The average of the maximum joint moments recorded from each trial was used for analysis.

### Statistical analysis

All data are presented as mean ± standard deviation, and α = 0.05. All analyses include all 22 participants (14 runners, 8 netballers), and all statistical tests were two-tailed. Data were first checked for normality using a Shapiro–Wilk test, and then an independent samples *t*-test was used to assess differences between groups (or a Mann–Whitney U test in the case of non-normally distributed data). Hedges’ *g* effect sizes with 95% confidence intervals were calculated to assess the magnitude of group differences and were interpreted as ≤ 0.2 = *trivial*, 0.2 to 0.5 = *small*, 0.5 to 0.8 = *medium*, and ≥ 0.8 = *large*. For tendon thicknesses, a 2 × 3 (group × region) mixed ANOVA was used to assess differences in tendon thickness between groups and measurement regions, as well group × region interaction effects. If significant main effects or interactions were found, Bonferroni *post-hoc* tests were used for pairwise comparisons. Pearson’s correlation coefficient (*r*) was used to assess the relationships between body mass and AT thickness and stiffness, where *r* ≤ 0.2 = *trivial*, 0.2 to 0.4 = *weak*, 0.4 to 0.6 = *moderate*, 0.6 to 0.8 = *strong*, 0.8 to 1.0 = *very strong*. Microsoft Excel (Microsoft Corporations, Redmond, WA, USA) and SPSS Statistics (IBM Corporations, Version 28.0, Armonk, NY, USA) were used for calculations and statistical analyses.

## Supplementary Information

Below is the link to the electronic supplementary material.

Supplementary Material 1

## Data Availability

The data generated and analysed for this study are available in Supplementary Information File 1.
